# The life expectancy of patients with metabolic syndrome after weight loss: study protocol for a randomized clinical trial (LIFEXPE-RT)

**DOI:** 10.1186/s13063-019-3304-9

**Published:** 2019-04-08

**Authors:** Oral Ospanov, Galymgan Eleuov, Irina Kadyrova, Farida Bekmurzinova

**Affiliations:** 1 Corporate Fund “University Medical Centre” (UMC), Kerey, Zhanibek khandar street 5/1, 010000 Nur-Sultan, Kazakhstan; 2 Surgical Department of the National Scientific Centre for Oncology and Transplantation, Kerey, Zhanibek khandar street 3, 010000 Nur-Sultan, Kazakhstan; 3 Department of Laparoscopic & Bariatric Surgery of Astana Medical University, Beybitshilik street 49A, 010000 Nur-Sultan, Kazakhstan

**Keywords:** Obesity, Bariatric, Metabolic syndrome, Life expectancy, Surgery, Weight loss therapy, Telomere length, Ospanov’s procedure, Gastric bypass, Band-separated gastric bypass

## Abstract

**Background:**

To date, surgeons and physicians have found positive results treating metabolic syndrome with surgical and non-surgical weight loss therapies. The purpose of this study was to evaluate changes in telomere length in patients with metabolic syndrome after weight loss.

**Methods/design:**

This study is a three-arm randomized controlled trial. The first group is composed of patients who have undergone stapleless bypass surgery (one anastomosis gastric bypass with an obstructive stapleless pouch and anastomosis (LOAGB-OSPAN)). The second group of patients underwent standard gastric bypass surgery (laparoscopic mini-gastric bypass-one anastomosis gastric bypass (LMGB-OAGB). The patients in the third group received non-surgical weight loss therapy, including a hypocaloric diet with energy restriction (− 500 kcal/day).

The aim is to compare changes—telomere length, body mass index, comorbidities, and quality of life—in patients with metabolic syndrome after weight loss.

**Discussion:**

To the best of our knowledge, this is the first randomized study to simultaneously compare the effects of surgical and non-surgical weight loss on changes in telomere length. It could provide a solution to the growing problem of metabolic syndrome. Normalization of the body mass index results in improvements in the health of patients with metabolic syndrome.

**Trial registration:**

ClinicalTrials.gov, NCT03667469. Registered on 11 September 2018.

**Electronic supplementary material:**

The online version of this article (10.1186/s13063-019-3304-9) contains supplementary material, which is available to authorized users.

## Background

The prevalence of obesity in the general population in Kazakhstan in 2017 was greater than 20%. The increase of the prevalence of obesity over the past 5 years was 3.9% [1]. Metabolic syndrome (MetS), which is the result of abdominal obesity, is a complex combination of symptoms that are risk factors for cardiovascular disease and manifestations of type 2 diabetes or prediabetes, non-alcoholic fatty liver disease, and dyslipidemia. Clinical MetS plays a leading role in reducing the life expectancy and increasing the mortality of Kazakhstan’s population [2].

Metabolic surgery should be recommended to treat type 2 diabetes in patients with class III obesity (BMI ≥ 40 kg/m2) and in those with class II obesity (BMI 35.0–39.9 kg/m^2^) when hyperglycemia is inadequately controlled by lifestyle and optimal medical therapy. Surgery should also be considered for patients with type 2 diabetes and BMI 30.0–34.9 kg/m^2^ if hyperglycemia is inadequately controlled despite optimal treatment with either oral or injectable medications. These BMI thresholds should be reduced by 2.5 kg/m^2^ for Asian patients [3].

Non-surgical weight loss therapies with an energy deficit of 500–1000 kcal/day should produce an approximately 10% body weight reduction over 6 months [4]. Reducing excess body weight positively affects the clinical course and life expectancy of patients with MetS [5]. Currently, surgeons and physicians have found positive results treating patients with MetS via surgical and non-surgical weight loss therapies [6]. The use of endoscopic staplers for surgical weight loss does not exclude the emergence of serious surgical complications, such as bleeding and leakage along the stapled suture line [7]. Therefore, the advantages of using a band in gastric bypass surgery justify its use from a safety point of view [8, 9].

It is known that increased systemic inflammation and oxidative stress associated with obesity can accelerate ageing, and telomere length (TL) can serve as an indicator of ageing at the cellular level [10]. Obesity has a known association with a shorter TL [11]. Weight loss in obese men is associated with increased telomere length [12]. However, the association of a reduction in body weight with a change in life expectancy remains unclear.

## Methods/design

### Study aim

The aim of this study was to evaluate the changes in telomere length in patients with MetS after weight loss induced by stapleless laparoscopic anastomosis gastric bypass–obstructive stapleless pouch and anastomosis (LOAGB-OSPAN), laparoscopic mini-gastric bypass–one anastomosis gastric bypass (LMGB-OAGB), and non-surgical weight loss therapy with energy restriction (− 500 kcal/day).

### Organization

The principal investigator (PI) is responsible for the overall project and for organizing steering committee meetings. An independent steering committee will be responsible for ensuring the overall safety of participants, coordinating study meetings, supervising the study, monitoring data safety, and overseeing the quality control.

Patient follow-up for all groups (1–3) will be carried out by the surgeon and endocrinologist and nutritionist.

### Study design

The study is designed as an interventional, prospective, randomized, controlled, single-center clinical trial. Patient enrolment started on May 24, 2018, and the last patient is expected to be included in the study on November 4, 2019. The study we be completed on September 30, 2020.

The ethics committee of the Corporate Fund “University Medical Centre” (UMC) has granted ethics approval for this study (May 24, 2018, approval number 5).

### Study population/participants and recruitment

Recruitment will be carried out by responsible bariatric surgeons with a minimum of 10 years of bariatric surgery experience in the Department of Surgery, National Scientific Center for Oncology and Transplantation (Astana, Kazakhstan). Screening will be performed on day − 7–0 prior to treatment to ensure that the patients fulfil the inclusion criteria. Patients will attend an informational meeting, at which they will be informed about the study purpose, process, and risk and benefits. Patients fulfilling the study criteria who sign the informed consent form will start treatment in accordance with the standard procedures of the trial site. Informed consent will be obtained from each participant by the investigators. During the trial, the investigators will continue to provide additional health care or compensation for participants’ health care needs that arise as a direct consequence of their participation in the trial.

### Eligibility criteria

The inclusion criteria are as follows:

1. Aged from 18 to 55 years.
2. BMI from 30 to 50 kg/m^2^.
3. Metabolic syndrome (MetS) with abdominal adiposity according to the presence of at least two of the following components of MetS: increased fasting plasma glucose levels detected before diabetes to pre-diabetes (HbA1 = 5.7–6.4 or a threefold increase in fasting plasma glucose > 5.6 mmol/l); previously diagnosed type 2 diabetes (HbA1 > 6.5 or glucose > 6.1); arterial hypertension (AD 130/85 mmHg or receiving antihypertensive therapy); increased triglyceride levels (> 1.7 mmol/L or receiving specific treatment for this disorder); decreased levels of high-density lipoprotein cholesterol (HDL-C < 1.03 mmol/L in men and < 1.29 mmol/L in women or receiving treatment for this disorder). The patient population will be included in the study if inadequately controlled despite optimal treatment for their diabetes, hypertension, and lipid disorders.
4. Available to receive treatment for 6 months, with the possibility of follow-up.
5. Provided written informed consent for randomization and treatment.

### Exclusion criteria

The exclusion criteria are as follows:

1. Aged less than 18 years or more than 55 years
2. BMI less than 30 kg/m^2^ or more than 50 kg/m^2^
3. Drug addiction or alcohol consumption
4. Complete immobilization of the patient (paresis/paralysis)
5. Presence in the anamnesis of a history of bariatric surgery
6. Insulin-dependent diabetes
7. Mental disorders or the use of antidepressants
8. Socially vulnerable categories (according to ethical principles)
9. Patients who do not understand the purpose of the study
10. Lack of informed written consent

### Withdrawal criteria

- If adverse events, especially severe adverse events, occur, researchers may consider withdrawal of patient(s) based on ethical and safety concerns.
- Patients drop out of the study.
- Patients voluntarily withdraw their informed consent.
- Serious violation of the study protocol by the subjects or investigators.
- Other reasons that the researchers believe are acceptable reasons for quitting the study.

### Randomization

Informed consent will be obtained from each participant before patient enrolment in the study. Patients who meet all the inclusion criteria and none of the exclusion criteria will be consecutively included and randomized into one of the three study arms by the study statistician, who is not involved in the enrolment, assignment, or assessment of patients, according to a random allocation sequence generated by Stata 7.0. The randomization list is kept strictly confidential. Allocation concealment is ensured with the use of sequentially numbered, identical, opaque, sealed envelopes (*n* = 60). The intervention will be communicated to the patient by a nurse who has no involvement in the enrolment or assessment of patients and who will open the sealed envelope during the visit before surgery.

- *Group 1 (A)*. The patients in Group 1 (*n* = 20) are treated by laparoscopic one anastomosis gastric bypass with an obstructive stapleless pouch and anastomosis (LOAGB-OSPAN).
- *Group 2 (B).* The patients in Group 2 (*n* = 20) are treated by laparoscopic mini-gastric bypass-one anastomosis gastric bypass (LMGB-OAGB) according to standard surgical procedures.
- *Group 3 (C).* The patients in Group 3 (*n* = 20) are treated by hypocaloric diet therapy with energy restriction. Standard diet for men and women of 1500 kcal/day − 500 kcal/day = 1000 kcal/day (an energy deficit of 500–1000 kcal/day).

### Blinding

In this study, the single-party independent evaluation method is used to evaluate the outcomes of the study. The outcome analyzer, study statistician, patients, and surgeons are blinded.

### General procedures and monitoring

#### Data collection and management

**Fig. 1:**
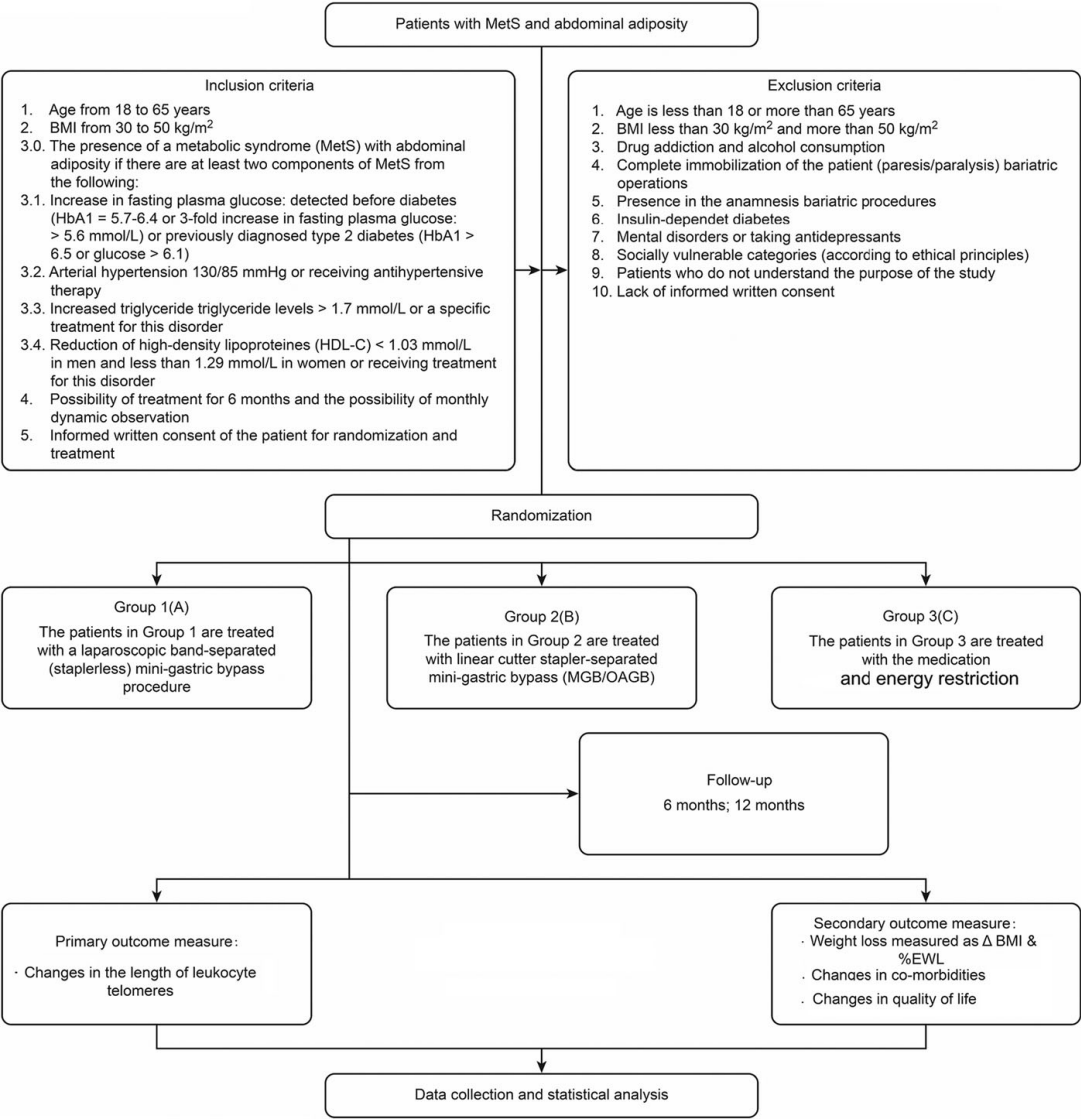
Study design (LIFEXPE-RT). *%EWL* percentage of excess weight loss

**Fig. 2:**
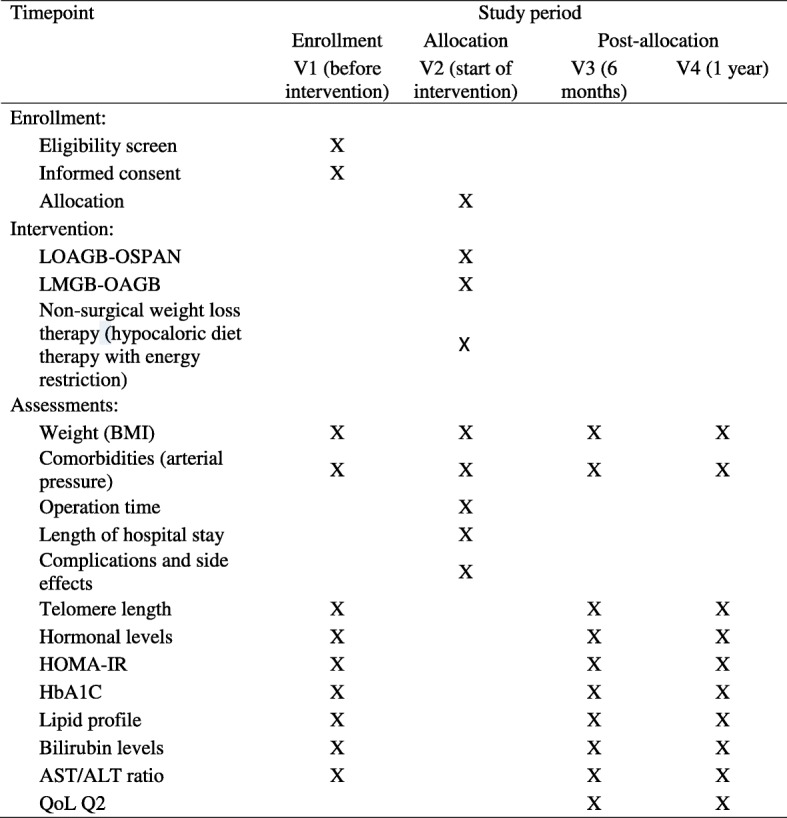
General procedure and monitoring process (flow diagram of the study design for the study entitled “A study of the life expectancy of patients with metabolic syndrome after weight loss: a comparative randomized controlled clinical trial”)

Treatment-related data are collected at V1 (before intervention) and at V2 (the start of the intervention or the baseline). According to the study protocol, follow-up data will be collected from V1 to months 6 (V3) and 12 (V4). Data collection begins on the day a participant signs the informed consent and continues until the termination of the trial or until the participant withdraws from the trial for any reason. If participants discontinue or deviate from the study protocols, the investigators will attempt to minimize the missing data. All original data are kept in chronological order for verification. Original data are transferred in a timely manner to a paper-based case report form (CRF) and an electronic database system located in a guarded facility at the trial site. Access to the study data is restricted. The PI will have access to the final dataset. An independent steering committee will monitor and examine adherence to the study protocol (Figs. 1 and 2).

### Outcome measures

#### Primary outcome measurement

1. Changes in the length of leukocyte telomeres

Changes in leukocyte TL will be determined in the patients in the three groups 6 and 12 months after surgery.

#### Secondary outcome measurements

2. Change in body mass index (**Δ** BMI)

This measurement assesses the change in BMI after the intervention. Weight (kg) and height (cm) will be combined in the BMI (kg/m^2^). The time frame is baseline, 6 months, and 12 months after surgery.

3. Changes in comorbidities

Changes in comorbidities will be assessed according to evaluation of the relevant symptoms and reported as the percentage of patients in whom there is an improvement in or resolution of diabetes, hyperlipidemia, hypertension, and obstructive sleep apnoea 6 and 12 months after surgery.

4. Changes in quality of life

Quality of life measured by the Moorehead-Ardelt questionnaire (Quality of Life Questionnaire II) 6 and 12 months after surgery.

### Statistical methods

#### Sample size

The sample size (*n* = 60) of this trial was estimated based on the literature and our own unpublished data.

### Data analysis

Normally distributed variables will be expressed as the mean and standard deviation (SD), and non-normally distributed variables will be expressed as the median and interquartile range; categorical variables will be expressed as the number and percentage (*n*, %). In test groups with continuous normally distributed variables, Student’s *t*-test will be used; the Mann–Whitney U test will be used for continuous non-normally distributed data. Categorical variables will be compared with the χ2 test or Fisher’s exact test; when appropriate, categorical variables will be reported as the relative risk. The statistical analyses will be conducted on an intention-to-treat basis. Multivariable analysis will be conducted by logistic regression and generalized mixed linear regression models with adjustment for any possible confounding covariates and with consideration of within-center variability. A *p* value of < 0.05 will be considered statistically significant.

### Populations for evaluation and missing data management

All evaluations, in particular the evaluation of the primary outcome measure, will be made with data from all randomized patients, regardless of whether they adhered to the treatment protocol or provided complete data sets. In particular, the following patients may be missing data:

- Those who discontinued the clinical trial will be evaluated as if they had completed the trial.
- Those whose planned examinations were not performed within the planned time frame will still be taken into consideration in the analysis.
- Patients who withdraw their consent to use their personal data for statistical analyses will be excluded from the analysis.
- Missing individual responses on the Quality of Life Questionnaire II will be replaced by simple imputation according to the recommendations in the test manual.

The reasons for any missing data will be analyzed, and any random missing data will be handled by the application of multiple imputation and model-based approaches, such as mixed models or weighted generalized estimating equations for repeatedly measured outcomes. Sensitivity analyses will be performed to examine the robustness of the results with reference to the assumptions made in the complete case analysis.

### Adverse events

An adverse event (AE) refers to any untoward event that occurs during the clinical study but that does not necessarily have a causal relationship with the surgical treatment. Safety evaluations are performed from the point at which the signature on the informed consent form is obtained until the end of the study or until the patient withdrawal from the trial, according to the management requirements. AEs and serious adverse events (SAEs) will be reported.

An SAE is an event that causes hospitalization, prolonged hospitalization, disability, incapacity, life-threatening illness or death, or congenital malformation during the clinical trial.

During the study, all AEs will be recorded. These records include the type of AE (using standard medical terminology), the date of the occurrence of the AE, the date of the disappearance/stabilization of the AE, the severity of the AE, the impact of the AE on the surgery, the relationship of the AE with the surgery, the treatment measures, and the outcomes. If an SAE occurs, researchers fill in an SAE report form. The report is signed and dated and reported to the ethics committee of the Corporate Fund University Medical Centre (UMC) and the clinical research centre of the National Scientific Centre for Oncology and Transplantation (Astana, Kazakhstan) within 24 h.

### Quality control

All surgeons and analyzers will be required to undergo special training prior to the trial to guarantee consistent practices. The training program will include information about diagnoses, inclusion/exclusion/withdrawal criteria, surgical techniques, follow-up procedures, and the completion of CRFs. The trial will be monitored by quality assurance personnel from the clinical research center of the National Scientific Center for Oncology and Transplantation, who will be independent from the study team, and an independent steering committee. Periodic monitoring will guarantee accuracy and quality throughout the study period. The essential documents (consent information, enrolment, protocol deviations, number and proportion of missed visits, and losses to follow-up) will be monitored and checked for accuracy and completeness by the monitors.

### Confidentiality

The relevant regulations of the data protection legislation will be maintained. All appropriate and necessary precautionary measures will be taken to ensure the confidentiality of the medical data and personal information. The safety of the data will be monitored by quality assurance personnel from the clinical research center of the National Scientific Center for Oncology and Transplantation, who will be independent from the study team, and an independent steering committee.

### Regulatory and ethical approval

The institutional review board of the Corporate Fund University Medical Center (UMC) has granted ethical approval for this study (May 24, 2018, approval number 5).

In the case of a necessary protocol amendment, the amendment will be submitted to the ethics committee and the quality assurance personnel from the Corporate Fund University Medical Center (UMC), who will be independent from the study team. Due to the study design (single-center, investigator-initiated trial) and the close contact between the study team and the site, a separate communication plan is not necessary.

## Discussion

At present, the results of scientific research have been published indicating that normalization of the BMI improves the health of patients with MetS [13]. It is important to determine the effect of a decrease in BMI on life expectancy through a randomized study.

No previous randomized study performing a simultaneous comparison of the association of surgical and non-surgical weight loss with changes in TL has been identified in the literature.

The aim of this study is to evaluate the change in telomere length in patients with MetS after weight loss due to stapleless band-separated gastric bypass, standard stapler-separated gastric bypass, and non-surgical weight loss therapy (Additional file 1).

## Trial registration

ClinicalTrials.gov, NCT03667469, registered on 11 September 2018.

## Trial status

At the time of the initial manuscript submission, recruitment had started (May 24, 2018), but it has not been completed. The last patient is expected to be included in the study on November 4, 2019.

## Additional file

Additional file 1: SPIRIT checklist. (PDF 169 kb)

## Abbreviations

AE: Adverse event
ALT: Alanine transaminase
AST: Aspartate transaminase
BMI: Body mass index
CRF: Case report form
GLP-1: Glucagon-like peptide
HbA1c: Glycated (glycosylated) hemoglobin
HDL: High-density lipoprotein
HOMA-IR: Homeostatic Model Assessment for Insulin Resistance
INR: International standard ratio
LMGB-OAGB: Laparoscopic mini-gastric bypass–one anastomosis gastric bypass
LOAGB-OSPAN: Laparoscopic one anastomosis gastric bypass–obstructive stapleless pouch and anastomosis
MetS: Metabolic syndrome
MGB: Mini-gastric bypass
OAGB: One anastomosis gastric bypass
QoL Q2: Quality of Life Questionnaire II
SAE: Serious adverse event
SD: Standard deviation
TL: Telomere length
UMC: University Medical Centre
