# Correction to: The life expectancy of patients with metabolic syndrome after weight loss: study protocol for a randomized clinical trial (LIFEXPE-RT)

**DOI:** 10.1186/s13063-019-3861-y

**Published:** 2019-12-12

**Authors:** Oral Ospanov, Galymzhan Yeleuov, Irina Kadyrova, Farida Bekmurzinova

**Affiliations:** 1 Corporate Fund “University Medical Centre” (UMC), Kerey, Zhanibek Khandar, Street 5/1, Nur-Sultan, Kazakhstan 010000; 2 Surgical Department of the National Scientific Centre for Oncology and Transplantation, Kerey, Zhanibek, Khandar Street 3, Nur-Sultan, Kazakhstan 010000; 3 Department of Laparoscopic & Bariatric Surgery of Astana Medical University, Beybitshilik, Street 49A, Nur-Sultan, Kazakhstan 010000

**Correction to: Trials**

**http://dx.doi.org/10.1186/s13063-019-3304-9**

Following publication of the original article [1], the authors notified us of a typing error in spelling Dr. Yeleuov’s name. The original publication has been corrected.

- Originally published name:
  Galymgan Eleuov
- Corrected name:
  Galymzhan Yeleuov
  The original article has been corrected.
