# Supplementary material for: The life expectancy of patients with metabolic syndrome after weight loss: study protocol for a randomized clinical trial (LIFEXPE-RT)
Source: Trials. 2019 Apr 8;20:202. doi: 10.1186/s13063-019-3304-9 (PMC6454761; doi:10.1186/s13063-019-3304-9)
Supplement: Supplementary file 1 — SPIRIT checklist. (PDF 169 kb) [file 13063_2019_3304_MOESM1_ESM.pdf]

SPIRIT 2013 Checklist: Recommended items to address in a clinical trial protocol and related documents*

Section/item	Item No	Description	Page Number on which item is reported
**Administrative information**			
Title	1	The life expectancy of patients with metabolic syndrome after weight loss: study protocol for a randomized clinical trial" (LIFEXPE-RT)	1
Trial registration	2a	ClinicalTrials.gov identifier: NCT03667469.	2
	2b		
Protocol version	3	September 11, 2018	2
Funding	4	Ministry of Education and Science of the Republic of Kazakhstan, grant number AP05135241.	1
Roles and responsibilitie s	5a	OO, IK and GE participated in the conception, design, and writing of the study protocol. IK contributed to the revision and editing of the study protocol. FB will be involved in the recruitment of patients and the acquisition of data. OO was involved in the critical revision of the manuscript. All authors approved the final version of the manuscript that has been submitted	17
	5b	Corporate Fund "University Medical Centre" (UMC)	1
	5c	The funder had no role in the study design, data collection, data analysis, data interpretation, or writing of the manuscript.	1
	5d		
**Introduction**			

Background and rationale	6a	The prevalence of obesity in the general population in Kazakhstan in 2017 was greater than 20%. The increase of the prevalence of obesity over the past five years was 3.9 percent. Metabolic syndrome (MetS), which is the result of abdominal obesity, is a complex combination of symptoms that are risk factors for cardiovascular disease and manifestations of type 2 diabetes or prediabetes, non-alcoholic fatty liver disease and dyslipidaemia. Clinical MetS plays a leading role in reducing the life expectancy and increasing the mortality of Kazakhstan's population. Metabolic surgery should be recommended to treat T2D in patients with class III obesity (BMI ≥40 kg/m2) and in those with class II obesity (BMI 35.0-39.9 kg/m2) when hyperglycemia is inadequately controlled by lifestyle and optimal medical therapy. Surgery should also be considered for patients with T2D and BMI 30.0-34.9 kg/m if hyperglycemia is inadequately controlled despite optimal treatment with either oral or injectable medications. These BMI thresholds should be reduced by 2.5 kg/m for Asian patients.<br>Reducing excess body weight positively affects the clinical course and life expectancy of patients with MetS. Currently, surgeons and physicians have found positive results treating patients with MetS via surgical and non-surgical weight loss therapies. The use of endoscopic staplers for surgical weight loss does not exclude the emergence of serious surgical complications, such as bleeding and leakage along the stapled suture line.	4
	6b	Group 1 (A). The patients in Group 1 (n=20) are treated by laparoscopic one anastomosis gastric bypass with an obstructive stapleless pouch and anastomosis (LOAGB-OSPAN).<br>Group 2 (B). The patients in Group 2 (n=20) are treated by laparoscopic mini- gastric bypass-one anastomosis gastric bypass (LMGB-OAGB) according to standard surgical procedures.<br>Group 3 (C). The patients in Group 3 (n=20) are treated by hypocaloric diet therapy with energy restriction. Standard diet for men and women of 1500 kcal/day -500 kcal/day = 1000 kcal/day (an energy deficit of 500-1000 kcal/day).	9
Objectives	7	The aim of this study was to evaluate the changes in telomere length in patients with MetS after weight loss induced by stapleless laparoscopic anastomosis gastric bypass – obstructive stapleless pouch and anastomosis (LOAGB-OSPAN), laparoscopic mini-gastric bypass- one anastomosis gastric bypass (LMGB-OAGB) and non-surgical weight loss therapy with energy restriction (-500 kcal/day).	5

Trial design	8	The study is designed as an interventional, prospective, randomized, controlled, single- centre clinical trial. Patient enrolment started on May 24, 2018, and the last patient is expected to be included in the study on November 4, 2019.	6
**Methods: Participants, interventions, and outcomes**			
Study setting	9	Academic hospital. National Scientific Centre for Oncology and Transplantation	6
Eligibility criteria	10	The inclusion criteria are as follows:<br>Age from 18 to 55 years;BMI from 30 to 50 kg/m^2;Metabolic syndrome (MetS) with abdominal adiposity according to the presence of at least two of the following components of MetS: increased fasting plasma glucose levels detected before diabetes to pre-diabetes (HbA1 = 5.7-6.4 or a 3-fold increase in fasting plasma glucose > 5.6 mmol/l); previously diagnosed type 2 diabetes (HbA1> 6.5 or glucose> 6.1); arterial hypertension (AD 130/85 mmHg or receiving antihypertensive therapy); increased triglyceride levels (> 1.7 mmol/L or receiving specific treatment for this disorder); decreased levels of high- 2<br>The exclusion criteria are as follows: high density lipoprotein cholesterol (HDL-C <1.03 mmol/L in men and < 1.29 mmol/L in women or receiving treatment for this disorder). The patient population will be included in the study if inadequately controlled despite optimal treatment for their diabetes, hypertension and lipid disorders. Available to receive treatment for 6 months, with the possibility of follow-up; 5. Provided written informed consent for randomization and treatment.	6
Interventions	11a	Group 1 (A). The patients in Group 1 (n=20) are treated by laparoscopic one anastomosis gastric bypass with an obstructive stapleless pouch and anastomosis (LOAGB-OSPAN).<br>Group 2 (B). The patients in Group 2 (n=20) are treated by laparoscopic mini- gastric bypass-one anastomosis gastric bypass (LMGB-OAGB) according to standard surgical procedures.<br>Group 3 (C). The patients in Group 3 (n=20) are treated by hypocaloric diet therapy with energy restriction. Standard diet for men and women of 1500 kcal/day -500 kcal/day = 1000 kcal/day (an energy deficit of 500-1000 kcal/day).	9

	11b	If adverse events, especially severe adverse events, occur, researchers may consider withdrawal of patient(s) based on ethical and safety concerns. Patients drop out of the study. Patients voluntarily withdraw their informed consent. Serious violation of the study protocol by the subjects or investigators. Other reasons that the researchers believe are acceptable reasons for quitting the study.	8
	11c	All surgeons and analyzers will be required to undergo special training prior to the trial to guarantee consistent practices. The training program will include information about diagnoses, inclusion/exclusion/withdrawal criteria, surgical techniques, follow-up procedures, and the completion of CRFs. The trial will be monitored by quality assurance personnel from the clinical research center of the National Scientific Center for Oncology and Transplantation, who will be independent from the study team, and an independent steering committee. Periodic monitoring will guarantee accuracy and quality throughout the study period. The essential documents (consent information, enrolment, protocol deviations, number and proportion of missed visits, and losses to follow-up) will be monitored and checked for accuracy and completeness by the monitors.	14
	11d		
Outcomes	12	Outcome measures Primary outcome measurement 1. Changes in the length of leukocyte telomeres Changes in leukocyte TL will be determined in the patients in the three groups 6 and 12 months after surgery. Secondary outcome measurements 2. Change in body mass index (Δ BMI) This measurement assesses the change in BMI after the intervention. Weight (kg) and height (cm) will be combined in the BMI (kg/m2). The time frame is baseline, 6 months, and 12 months after surgery. 3. Changes in comorbidities Changes in comorbidities will be assessed according to evaluation of the relevant symptoms and reported as the percentage of patients in whom there is an improvement in or resolution of diabetes, hyperlipidemia, hypertension, and obstructive sleep apnoea 6 and 12 months after surgery. 4. Changes in quality of life. Quality of life measured by the Moorehead-Ardelt questionnaire (Quality of Life Questionnaire II) 6 and 12 months after surgery.	10

Participant timeline	13	An independent steering committee will monitor and examine adherence to the study protocol (Figs. 1 and 2).	21-22
Sample size	14	The sample size (n = 60) of this trial was estimated based on the literature and our own unpublished data.	11
Recruitment	15	Recruitment will be carried out by responsible bariatric surgeons with a minimum of 10 years of bariatric surgery experience in the Department of Surgery, National Scientific Center for Oncology and Transplantation (Astana, Kazakhstan). Screening will be performed on day − 7–0 prior to treatment to ensure that the patients fulfil the inclusion criteria. Patients will attend an informational meeting, at which they will be informed about the study purpose, process, and risk and benefits. Patients fulfilling the study criteria who sign the informed consent form will start treatment in accordance with the standard procedures of the trial site. Informed consent will be obtained from each participant by the investigators. During the trial, the investigators will continue to provide additional health care or compensation for participants' health care needs that arise as a direct consequence of their participation in the trial.	6
**Methods: Assignment of interventions (for controlled trials)**			
Allocation:			
Sequence generation	16a	The intervention will be communicated to the patient by a nurse	8
Allocation concealment mechanism	16b	Allocation concealment is ensured with the use of sequentially numbered, identical, opaque, sealed envelopes (n = 60).	8
Implementation	16c	A nurse has no involvement in the enrolment or assessment of patients and who will open the sealed envelope during the visit before surgery	8
Blinding (masking)	17a	In this study, the single-party independent evaluation method is used to evaluate the outcomes of the study. The outcome analyzer, study statistician, patients, and surgeons are blinded.	9

	17b	An adverse event refers to any untoward event that occurs during the clinical study but that does not necessarily have a causal relationship with the surgical treatment. Safety evaluations are performed from the point at which the signature on the informed consent form is obtained until the end of the study or until the patient withdrawal from the trial, according to the management requirements	13
**Methods: Data collection, management, and analysis**			
Data collection methods	18a	Treatment-related data are collected at V1 (before intervention) and at V2 (the start of the intervention or the baseline). According to the study protocol, follow-up data will be collected from V1 to months 6 (V3) and 12 (V4). Data collection begins on the day a participant signs the informed consent and continues until the termination of the trial or until the participant withdraws from the trial for any reason.	9
	18b		
Data management	19	If participants discontinue or deviate from the study protocols, the investigators will attempt to minimize the missing data. All original data are kept in chronological order for verification. Original data are transferred in a timely manner to a paper-based case report form (CRF) and an electronic database system located in a guarded facility at the trial site. Access to the study data is restricted. The PI will have access to the final dataset.	9-10
Statistical methods	20a	Normally distributed variables will be expressed as the mean and standard deviation (SD), and non-normally distributed variables will be expressed as the median and interquartile range; categorical variables will be expressed as the number and percentage (n, %). In test groups with continuous normally distributed variables, Student's t-test will be used; the Mann–Whitney U test will be used for continuous non-normally distributed data. Categorical variables will be compared with the $\chi 2$ test or Fisher's exact test; when appropriate, categorical variables will be reported as the relative risk. The statistical analyses will be conducted on an intention-to-treat basis. Multivariable analysis will be conducted by logistic regression and generalized mixed linear regression models with adjustment for any possible confounding covariates and with consideration of within-center variability. A p value of < 0.05 will be considered statistically significant.	11-12
	20b		

	20c	All evaluations, in particular the evaluation of the primary outcome measure, will be made with data from all randomized patients, regardless of whether they adhered to the treatment protocol or provided complete data sets. In particular, the following patients may be missing data: Those who discontinued the clinical trial will be evaluated as if they had completed the trial. Those whose planned examinations were not performed within the planned time frame will still be taken into consideration in the analysis. Patients who withdraw their consent to use their personal data for statistical analyses will be excluded from the analysis.	12-13
**Methods: Monitoring**			
Data monitoring	21a	An independent steering committee will monitor and examine adherence to the study protocol	9
	21b	The PI will have access to the final dataset.	10
Harms	22	An adverse event (AE) refers to any untoward event that occurs during the clinical study but that does not necessarily have a causal relationship with the surgical treatment. Safety evaluations are performed from the point at which the signature on the informed consent form is obtained until the end of the study or until the patient withdrawal from the trial, according to the management requirements. AEs and serious adverse events (SAEs) will be reported.	13
Auditing	23	The trial will be monitored by quality assurance personnel from the clinical research center of the National Scientific Center for Oncology and Transplantation, who will be independent from the study team, and an independent steering committee. Periodic monitoring will guarantee accuracy and quality throughout the study period. The essential documents (consent information, enrolment, protocol deviations, number and proportion of missed visits, and losses to follow-up) will be monitored and checked for accuracy and completeness by the monitors.	14
**Ethics and dissemination**			
Research ethics approval	24	The ethics committee of the Corporate Fund "University Medical Centre" (UMC) has granted ethical approval for this study (May 24, 2018, approval 5).	18

Protocol amendments	25	In the case of a necessary protocol amendment, the amendment will be submitted to the ethics committee and the quality assurance personnel from the Corporate Fund University Medical Center (UMC), who will be independent from the study team. Due to the study design (single-center, investigator-initiated trial) and the close contact between the study team and the site, a separate communication plan is not necessary.	15
Consent or assent	26a	Not applicable. The results will be presented at relevant national and international conferences and will be published in peer-reviewed journals	18
	26b		
Confidentiality	27	The relevant regulations of the data protection legislation will be maintained. All appropriate and necessary precautionary measures will be taken to ensure the confidentiality of the medical data and personal information. The safety of the data will be monitored by quality assurance personnel from the clinical research center of the National Scientific Center for Oncology and Transplantation, who will be independent from the study team, and an independent steering committee.	14
Declaration of interests	28	The authors declare that they have no competing interests.	18
Access to data	29	Access to the study data is restricted. The PI will have access to the final dataset.	10
Ancillary and post-trial care	30	During the trial, the investigators will continue to provide additional health care or compensation for participants' health care needs that arise as a direct consequence of their participation in the trial.	6
Dissemination policy	31a	The datasets generated or analyzed during the current study are available from the corresponding author upon reasonable request.	17
	31b		
	31c		
**Appendices**			
Informed consent materials	32	Patients fulfilling the study criteria who sign the informed consent form will start treatment in accordance with the standard procedures of the trial site. Informed consent will be obtained from each participant by the investigators.	6

Biological specimens	33	Not applicable.	

*It is strongly recommended that this checklist be read in conjunction with the SPIRIT 2013 Explanation & Elaboration for important clarification on the items. Amendments to the protocol should be tracked and dated. The SPIRIT checklist is copyrighted by the SPIRIT Group under the Creative Commons "Attribution-NonCommercial-NoDerivs 3.0 Unported" license.
